# A rapid decline in gender bias relates to changes in subsistence practices over demographic changes in a formerly matrilineal community

**DOI:** 10.1016/j.isci.2025.111926

**Published:** 2025-01-31

**Authors:** Yaming Huang, Pengpeng Bai, Liqiong Zhou, Ruth Mace, Juan Du

**Affiliations:** 1 State Key Laboratory of Grassland and Agro-Ecosystems, College of Ecology, Lanzhou University, 222 Tianshui South Road, Lanzhou, Gansu Province 730000, P.R. China; 2 Department of Anthropology, University College London, 14 Taviton Street, London WC1H 0BW, UK; 3 Institute of Advanced Study at Toulouse (IAST), Toulouse School of Economics, Toulouse, France

**Keywords:** Social sciences, Anthropology

## Abstract

This research examines dynamics of kinship systems, emphasizing changes in gender-biased inheritance and social interaction within a formerly matrilineal community. Using demographic data over 70-year of lifespan from 17 Tibetan villages, we observe a significant shift within the predominantly matrilineal inheritance structure: a once-prevalent preference for females in older cohorts has now gone in recent generations. We explore two possible explanations: that this is driven by changes in subsistence system or by changes in sibling configuration. Our investigation reveals that a change from agriculture to non-traditional economy with more market integration marks a pivot from matrilineal to non-unilineal inheritance systems. Moreover, results from economic games conducted in two distinct survey periods (2015 and 2021) indicate that high donations for females in 2015 have become unbiased in 2021. These findings provide concrete evidence of shifts in gender preference both at the level of familial resource allocation and broader societal interactions.

## Highlights

• Agriculture favors women while pastoralism prefers men
• Demographic shifts from female-biased inheritance systems to a more gender-neutral framework
• Social support has also transitioned from a female bias to a more gender-neutral approach
• Subsistence strategies rather than demographic transitions drive this change

## Graphical abstract

**Figure:**
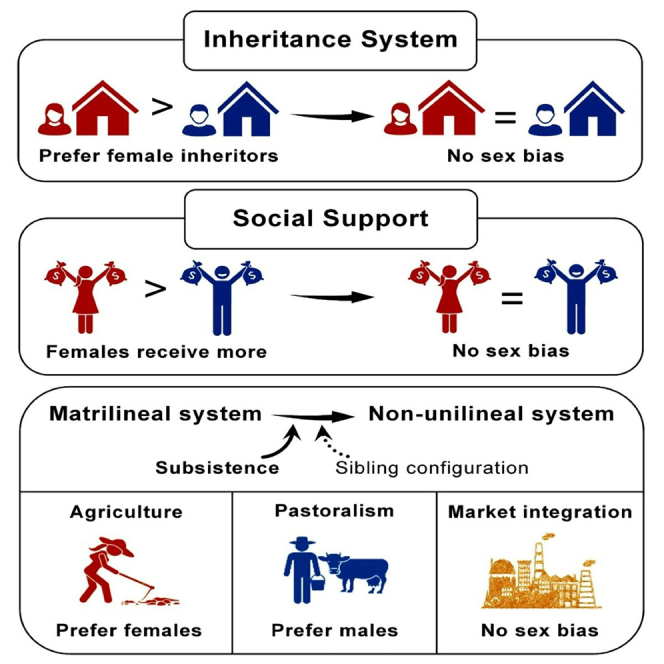

## Introduction

Anthropological descent systems establish the principles of kinship based on parent-child relationships, mostly categorized into patrilineal or matrilineal systems, which focus on lineage through the father or mother, respectively.^1^

Inheritance pattern lies at the heart of descent system that significantly shapes dispersal practices or post-marital residence patterns.^2^ In patrilineal societies, patrilocal (or virilocal) residence pattern is generally dominant—where females disperse after marriage and males stay in their natal areas. Sons are prioritized for the allocation of intergenerational assets, as widely observed across various global regions including Bangladesh, Bengaluru, Gambia, and Finland.^3^^,^^4^^,^^5^^,^^6^ Conversely, matrilineal systems, evident in Chewa, Khasi, and Broken K Pueblo communities, generally favor daughters with female philopatry.^7^^,^^8^^,^^9^^,^^10^ Exceptions exist, such as some African Bantu societies where matrilineal-related males inherit property,^11^ whereas in some Tibetan patrilineal societies, females receive greater parental investment.^12^

Descent system profoundly impacts both the distribution of family resources and broader gender dynamics, influencing negotiation between sexes.^13^ Ethnographic records often note increasing autonomy for women in matrilineal societies.^14^ Evolutionary perspectives posit that matrilineal societies generally exhibit less gender disparities compared to their patrilineal counterparts, despite prevalent male dominance.^15^^,^^16^^,^^17^ This differentiation is stark in domestic settings where virilocality aligns with husband dominance, while matrilineal setups usually show a more balanced power distribution.^15^ Other characteristics of matrilineal societies include higher divorce rates and greater sexual autonomy for women.^16^^,^^18^ A recent study conducted by Scelza et al., associating inheritance preferences with female autonomy, shows a negative correlation between patrilineal preferences and permissiveness for women.^19^ Chen et al. reveal the association between post-marital dispersal and workload, indicating that the dispersing sex often shoulders a heavier workload.^20^

More broadly, kinship system, which affects gender roles, rights, and responsibilities within a society, is intricately linked with gender status and shape the strategies each gender employs in constructing social relationships.^21^ Commonly, “universal gender differences” hypothesis posits that strategies of men and women utilizing social networks that are consistent across genders, despite varying social structures.^22^ Men’s reproductive success is primarily constrained by their ability to secure reproductive partners, whereas women’s reproductive success is predominantly limited by the availability of resources necessary for children’s survival and development.^23^ Women primarily commit to parental investment through pregnancy, lactation, and childcare, and are hypothesized to put more efforts into forming and sustaining relationships that support these responsibilities.^24^ In contrast, men are typically expected to use social relationships to achieve status-related goals that increase their mating opportunities.^25^ Therefore, women are anticipated to maintain closer and more stable relationships, whereas men are expected to develop broader social networks with more tenuous connections.^26^ However, the “gender reversal” hypothesis suggests that in specific social settings, such as matrilineal societies, traditional gender differences are minimized or even reversed, leading to women’s social networks resembling those typically seen in men.^21^ In matrilineal societies, gender-specific social interaction strategies can differ significantly from those in patrilineal societies.^27^ Recent research, comparing a matrilineal society with a patrilineal one, found that in the patrilineal society, men have larger and more central social networks while the reverse is true in the matrilineal society, supporting the hypothesis that gender-specific social strategies vary depending on societal contexts.^28^

Matrilineal system has always been relatively rare and believed to be declining.^11^^,^^29^^,^^30^ The female-biased kinship system accounts for only about 17% of surveyed societies, significantly fewer than the proportion of patrilineal societies (about 41%).^31^ The rarity and persisted decreasing of matrilineal arrangements were historically attributed to their perceived instability, stemming from conflicting evolutionary interests between genders, which influenced distinct gender roles within social groups.^11^^,^^23^^,^^24^ Commonly, gender roles are not easily interchangeable, with continuous gender-based labor inequality due to both physical demands and enduring cultural norms.^20^^,^^32^ Global studies show a considerable gender gap in property ownership, with men typically owning more property than women.^33^ Additionally, sex bias influences competition dynamics: men commonly compete for resources with non-kin males and receive more communal supports, while women’s competition tends to be confined within familial contexts.^34^ Consequently, gender bias against women is observed in both men and women, leading to the emergence of “boys’ clubs.”^34^ Contemporary evolutionary theory, from the perspective of individual motivation and trade-offs between benefits and costs of fitness, focuses on how diverse socio-ecological factors shape the roles of men and women, and ultimately establish women as central in families and/or societies, rather than regarding a decline of matrilineal systems as universal.^8^^,^^35^

Human societies are profoundly shaped by parental investment and socio-economic dynamics.^8^^,^^36^^,^^37^^,^^38^ Parent-offspring conflict highlights that the nature of parental investment is influenced by constraints, meaning some children may be treated better than others.^39^^,^^40^ Parental investment varies with socio-economic dynamics, as a recent study indicates that following the 2008–2009 financial crisis, women in rural Greece shifted their investment in grandchildren from a patrilateral to a matrilateral bias.^41^ Sibling competition for limited resource acquisition is intense, and sibling configuration significantly affects the degree of parental investment on each child.^42^ For instance, in societies where inter-generational resources are transferred along the maternal line, females often experience intense competition among themselves for inheritance benefits or dowry.^8^^,^^43^ Conversely, in the predominantly patrilineal Arsi region, land inheritance is closely linked to the number of elder brothers, intensifying competition with each additional male sibling.^44^

Kinship systems are also deeply connected to subsistence system that defines sexual division of labor and broader societal structures.^2^^,^^45^ Traditional egalitarian hunter-gatherer societies practiced equitable resource sharing.^46^^,^^47^ Some argue the introduction of ploughing agricultural practices or intensive animal husbandry has shifted many societies from matrilineal to patrilineal structures.^48^ This change is evident in various regions, with the spread of cattle in sub-Saharan Africa causing a decline of matriliny.^17^ While in some Pacific areas, specialized fishing practices that increase male absences have strengthened matriliny.^49^ Market integration has introduced new dynamics.^16^^,^^18^ The economic globalization, with the shift of productive types from traditional to market-driven patterns, presents both challenges and opportunities for matriliny.^50^^,^^51^ Some contend that as economy modernizes, women’s autonomy is at risk, largely because industrialization can inadvertently perpetuate gender inequalities.^52^ Conversely, others argue that integration into global markets has the potential to enhance women’s influence and access to resources.^53^^,^^54^ This dichotomy is clear in China, where tourism-driven market activities have led to divergent outcomes for matrilineal and patrilineal villages within the same regions.^55^^,^^56^ He and Wu, using data from the 2005 population mini-census, reported that marketization exacerbated gender earnings inequality in urban China’s labor markets.^57^ However, Chen et al. found foreign participation and export orientation significantly boost female employment, underscoring the role of globalization in reducing gender discrimination.^58^

The decline of matrilineal societies is well-recognized, though the bulk of evidence comes from phylogenetic analyses and ethnographic observations.^17^^,^^30^^,^^59^^,^^60^^,^^61^ Empirical research into societies undergoing this shift remains limited. Considering the context-dependence social evolution, our study seeks to empirically outline the demographic shifts in kinship system with a focus on gender-specific inheritance system. We investigate a formerly matrilineal population using a comprehensive demographic dataset reporting over 70 years, covering 3,836 individuals. Our ongoing research aims to uncover the fundamental socio-ecological factors linked to transitions in descent systems, focusing on sibling configuration and subsistence strategy. Additionally, we investigated whether changes in gender bias within inheritance systems result in corresponding shifts in gender bias at the community level, particularly in social interactions, by conducting economic games across two distinct survey periods.

### (H1) Sibling configuration

Implementation of family planning policies and observations of declining fertility in our study site suggest demographic changes in sibling configurations, such as a decrease in the number of brothers or sisters or an increase in the possibility that parents may not have a son or a daughter. We thus hypothesize that demographic shifts in sibling structure are linked to demographic changes in gender bias within the inheritance system.

### (H2) Subsistence system

In our study site, as an increasing portion of family income comes from market-oriented activities, the redistribution of gender roles has impacted the intersexual competition for familial resources. We thus hypothesize that demographic shifts in subsistence system are related to demographic changes in gender bias within the inheritance system.

### (H3) Gender roles in social interactions

Gender-biased social interactions are influenced by the evolving inheritance system. Inheritance system determines which gender receives more social supports in alignment with which gender is allocated the majority of family resources, reflected in the variations of gender differences in economic games across different survey years.

## Results

### Inheritance system

**Figure 1:**
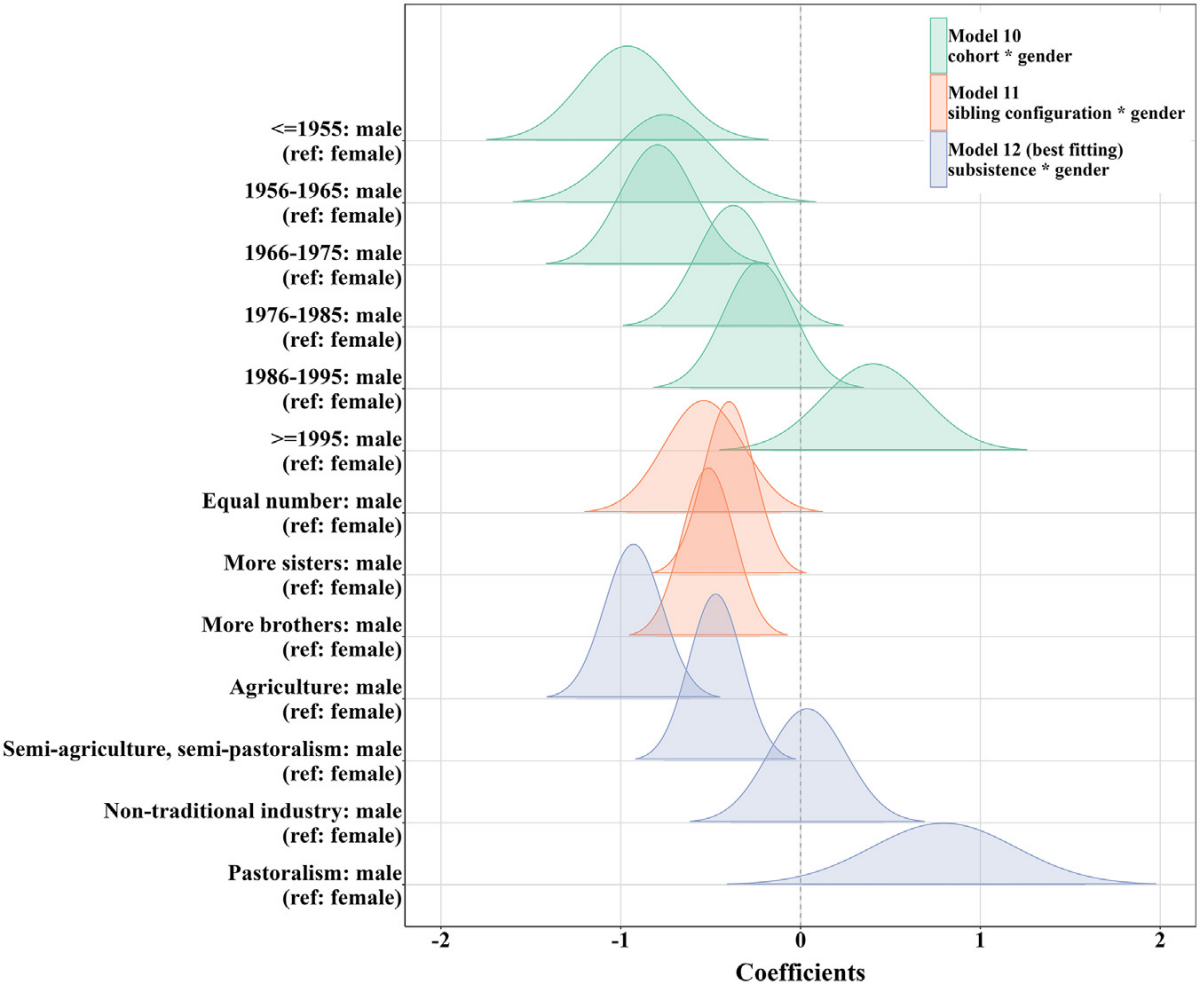
Coefficient estimates of gender difference in inheriting family wealth Distribution figures are plotted for fixing cohorts (green), sibling configuration (orange), and subsistence system (purple), respectively.

**Figure 2:**
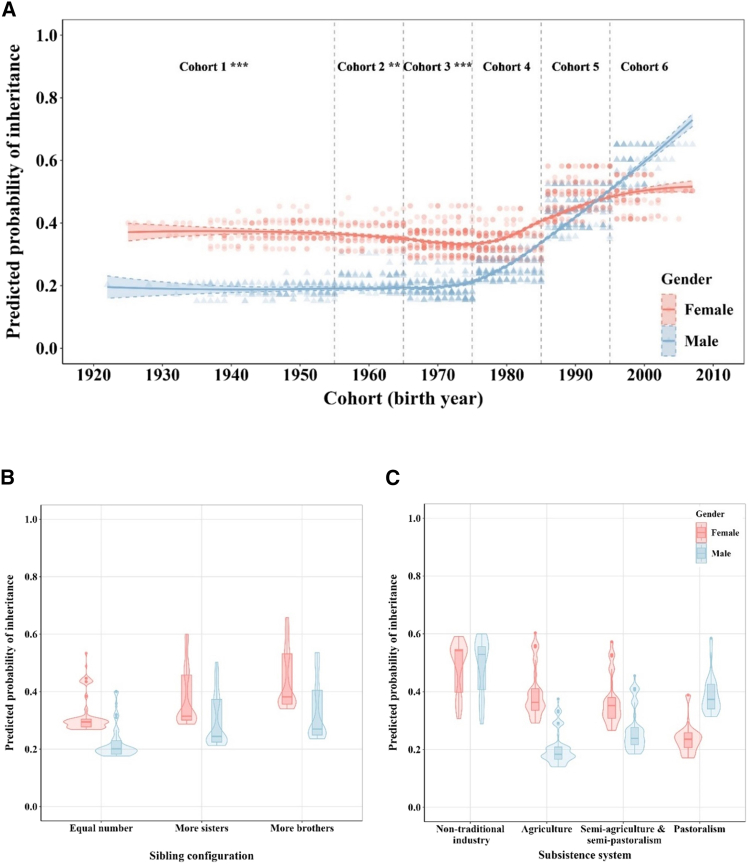
Predicted probabilities of inheriting family wealth (A) The interaction effects of gender and birth cohorts (divided by 10-year intervals) from model 10. (B) The interaction effects of gender and sibling configuration from model 11. (C) The interaction effects of gender and subsistence system from model 12. Red represents females and blue represents males.

Our demographic data highlight a shift over time in the sex-biased inheritance system, with the decreasing advantages for females becoming inheritors. Among people born before 1985, males have a lower probability of inheriting parental wealth compared to females (model 10: OR_≤1955_ = 0.38, *p*_≤1955_ < 0.001; OR_1956–1965_ = 0.47, *p*_1956–1965_ = 0.007; OR_1966–1975_ = 0.45, *p*_1966–1975_ < 0.001 and OR_1976–1985_ = 0.69, *p*_1976–1985_ = 0.067) (Figure 1; Table S6). However, this advantage has decreased over time, leading to a scenario in recent cohorts where there is no significant difference in inheritance probabilities between genders (model 10: OR_1986–1995_ = 0.79, *p*_1986–1995_ = 0.23 and OR_>1995_ = 1.50, *p*_>1995_ = 0.16) (Figure 1; Table S6). In Figure 2A, the inter-sexual difference in inheriting parental wealth begins diminishing after about 1975, earlier than the timing of beginning implementing fertility policies (1979), and disappears after about 1990, consistent with the timing of strictly implementing fertility policies by the local government (1991).

**Figure 3:**
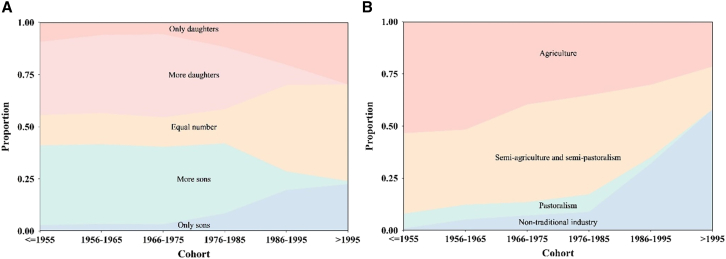
Proportion of offspring configurations and main household livelihood form by birth cohorts (A) Red represents only daughters, pink represents more daughters, yellow represents equal number of sons and daughters, green represents more sons and blue represents only sons. (B) Red represents agriculture, yellow represents semi-agriculture and semi-pastoralism, green represents pastoralism, and blue represents non-traditional industry (see also Methods S1).

Further investigation into whether sibling configuration (H1) or subsistence system (H2) correlates with these demographic changes of gender bias in inheritance system. The findings challenge H1 by demonstrating that gender differences in competitive ability for inheriting parental resources are consistence across various sibling configurations, although there are demographic changes in sibling configuration (Figure 3A). The results show that females consistently have a higher likelihood of securing family resources than males, regardless of whether they have more sisters, or more brothers or non-biased sibling configuration (model 11: OR _equal number_ = 0.58, *p*_equal number_ = 0.015; OR _more sisters_ = 0.67, *p*_more sisters_ = 0.005 and OR _more brothers_ = 0.60, *p* _more brothers_ < 0.001) (Figures 1 and 2B; Table S7). After including the interaction between gender and sibling configuration, there are not significant differences between model 10 and model 13, which further proves the notion that demographic shifts in sibling configurations are not strongly associated with demographic shifts in gender bias within inheritance system (Tables S5 and S6). Moreover, our results indicate that individuals, irrespective of their gender, are in a more advantageous position to inherit parental assets when they have more brothers (Figure 2B; Table S5). This suggests that the presence of more brothers within a family tends to reduce the competition for inheritance among siblings.

Our results provide direct evidence that subsistence system (H2) plays a crucial role in influencing gender disparities for parental resources. Specifically, agriculture provides a marked advantage to women in inheriting family wealth (model 12: OR = 0.40, *p* < 0.001) (Figures 1 and 2C; Table S8). This advantage persists, albeit diminishes, when the subsistence strategy shifts to a mixed pattern, such as in semi-agriculture and semi-pastoralism, where the female advantage in competing for parental resources decreases (model 12: OR = 0.62, *p* = 0.002) (Figures 1 and 2C; Table S8). Conversely, pastoralism reverses this trend, favoring males as inheritors (model 12: OR = 2.22, *p* = 0.047) (Figures 1 and 2C; Table S8). Moreover, when non-traditional industry is the main source of family income, there is not significant gender difference (model 12: OR = 1.04, *p* = 0.86) (Figures 1 and 2C; Table S8). In summary, traditional production forms are accompanied with gender roles, with women gaining more from agricultural activities and men benefiting more from pastoralism. However, non-traditional forms of production activities that integrate markets erode gender disparities. This study also observes generational changes in family income sources, illustrating a significant shift from traditional livelihoods, initially agriculture followed by pastoralism, to non-traditional livelihoods (Figure 3B). In the oldest cohort (≤1955), more than 50% of families depend primarily on agriculture, followed by semi-agricultural and semi-pastoral form at about 38%. Over time, there has been a noticeable shift away from these traditional subsistence strategies toward economic activities such as business. In the most recent cohort, more than half of the families derive their main income from non-traditional sectors. Based on ethnographic data between 2000 and 2015 from China Yearbook Database, we also observe a similar tendency in economic development (Table S12; Figure S3). At the turn of the 21st century, pastoralism was the predominant economic driver, accounting for approximately 50% of income, while agriculture and non-traditional industries each fluctuated around 20%. By 2015, non-traditional industries had ascended to become the main economic contributors, accounting for about 50% of income as pastoralism waned. It is obvious that agriculture had already been in a decline, which had been overtaken by pastoralism as the primary sector earlier in the century. Post-2010, the non-traditional sector emerged as the dominant income source. Empirical data from household surveys conducted in 2015 and 2021 further confirm that in 2015, household income mainly comes from non-traditional industries, accounting for about 70% (Figure S4). In 2021, the proportion of household income from non-traditional industries further increases, exceeding 85%. These two lines of evidence (ethnographic and empirical data) highlight the rapid development and eventual dominance of non-traditional income sources, supplanting traditional economic activities, both agriculture and pastoralism. These trends in subsistence system covary with demographic shifts in gender bias within inheritance dynamics.

Comparisons among model 10, model 14 and model 15 do straightforwardly prove that demographic changes of sex-biased inheritance system mostly result from subsistence-specific gender difference (Table S6) and demographic changes in subsistence strategies (ethnographic and empirical information presented previously). Our results provide a clear linkage to changing patterns of gendered inequality in inheritance practices, supporting hypothesis of subsistence system (H2). The transformation from traditional agriculture and pastoralism to a predominantly non-traditional industrial economy is closely linked with shifts in the demographic patterns of sex-biased inheritance system.

### Gifts received

In study-1 (2015), a total of 188 participants nominated 148 recipients, whereas in study-2 (2021), 73 participants nominated a total of 103 recipients (see Table S3). This discrepancy suggests that participants in study-1 (2015) likely had denser social networks outside their households than those in study-2 (2021). Furthermore, the average gift unit in study-1 (2015) is 1.52 ± 2.61, with recipients receiving an average of 17.60 ± 37.57 yuan (Table S3). In contrast, in study-2 (2021), the average gift unit is 1.21 ± 0.7, and the average amount of money is 9.36 ± 8.57 yuan (Table S3). Consequently, recipients in study-1 (2015) generally gained more from gift games in terms of both the number of gifts and the monetary value than those in study-2 (2021).

**Figure 4:**
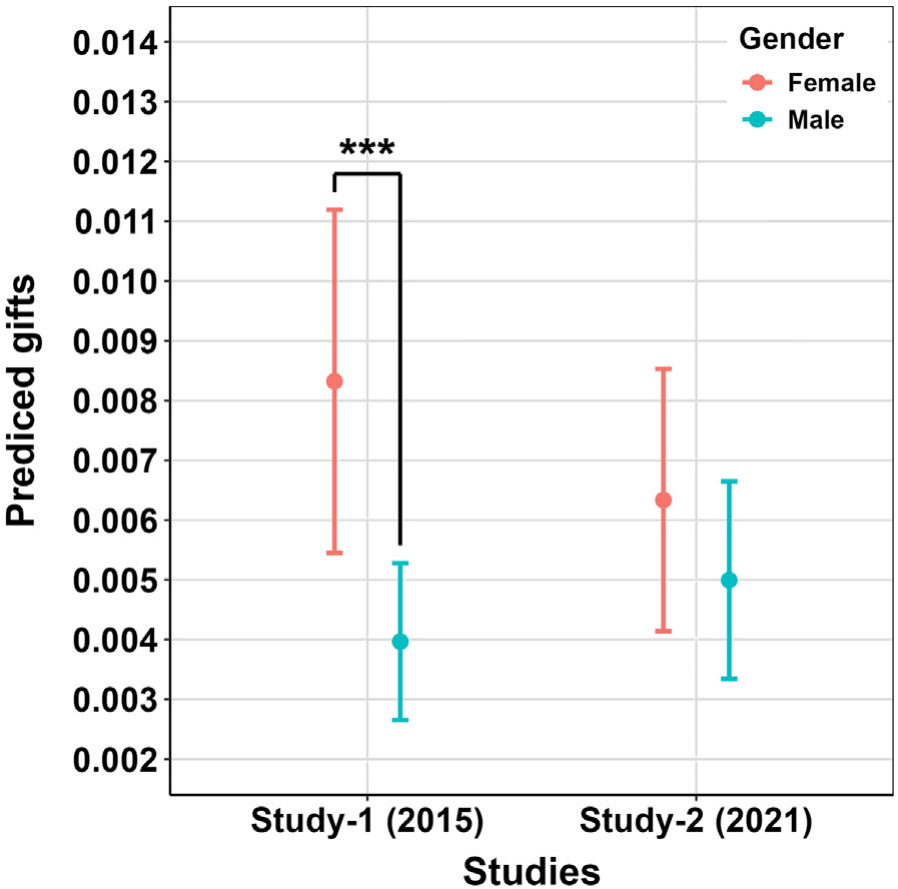
Predicted gifts receiving in dyads (ego—alter pairs) by study time and gender of recipients *n* = 260 individual with *dyad* = 586,544 social relationship information is used in this plot. The data are predicted from model 10 about gift received. Data are represented as mean ± SD. Red point and error bar represent female, while blue point and error bar represent male. ∗∗∗ represents *p* < 0.001.

Our findings reveal variations in how gender influences social dynamics over different study periods. In study-1 (2015), women have a notable advantage over men in receiving gifts (IRR = 0.50, *p* < 0.001) (Figure 4; Table S10). Conversely, in study-2 (2021), there is no significant inter-sexual difference in terms of being beneficiaries in gift-received games (IRR = 0.83, *p* = 0.37) (Figure 4; Tables 1 and S10). It appears that while gender differences in inheritance system began changing around 1975, notable gendered difference in social interactions remained persisted in 2015 and only disappeared afterward. These observations support the hypothesis that shifts in gender-specific social interactions align with demographic changes in gender bias within inheritance system, varying from a female preference to a neutral stance (supporting H3).

**Table 1:** Generalized estimating equations (Poisson) predicting gifts received in dyads (ego—alter pairs) within the same township

Variable	IRR	95% CI	*P*
Intercept	0.004	0.003–0.006	<0.001∗∗∗
Ego’s age	1.00	0.89**–**1.12	0.99
Ego’s dispersal pattern (ref: dispersal)	0.97	0.74–1.26	0.80
Ego’s gender (ref: female)	0.98	0.77–1.26	0.91
Alter’s age^a^	1.25	1.11–1.42	<0.001∗∗∗
Alter’s dispersal pattern (ref: dispersal)^a^	1.70	1.29–2.25	<0.001∗∗∗
Alter’s gender × time (ref: female: 2021)			
Male: 2021	0.83	0.56–1.24	0.37
Female: 2015	1.33	0.96–1.85	0.09
Male: 2015^a^	0.60	0.36–0.99	0.044∗

*n*_*all*_ = 260, *dyad*_*all*_ = 586,544.

a Statistical significance. ∗*p* < 0.05, ∗∗*p* < 0.01, ∗∗∗*p* < 0.001.

## Discussion

Our research, analyzing demographic data spanning over 70 years, covering 3,836 individuals’ detailed demographical information from 17 Tibetan villages, reveals a significant shift from a female-biased inheritance system to a more gender-neutral framework (H1). The decline of matrilineal system has been evident from phylogenetic analyses and ethnographic observations in cross-cultural comparisons.^30^^,^^31^^,^^61^ However, there is a scarcity of long-term empirical research with a focus on a certain region, which this study aims to address. This demographical shift of gender preference is evident in the possibility of individuals to remain in their natal families and inherit a substantial portion of family wealth. The decision about dispersing or staying in one’s birthplace is shaped by the costs and benefits of cooperation and competition between an individual’s own interests and those of their co-residing kin.^37^ Likewise, gender difference in the cost and benefit of fitness lead to skewed parental investments, resulting in some children receiving better treatments than others.^23^ Furthermore, the family planning policy has been advocated since 1979 and become more strict since 1991.^62^ Restrictive fertility policies directly limit the number of births (Figure 3A), thereby influencing sibling configurations.^42^ However, our results indicate that females consistently have a higher likelihood of securing the right to inherit family resources than males, irrespective of having more sisters, more brothers, or a balanced sibling configuration. Although there are clearly demographic changes in sibling configurations, varying sibling setups do not affect women’s absolute advantage in competing for inheritance rights. Therefore, our results do not support the association between demographic shifts in gender bias within inheritance system and demographic shifts in sibling configuration (H2).

Kinship systems are closely linked to subsistence methods, which shape broader societal structures.^2^^,^^45^ The relationship between gender bias and inheritance systems is intricately tied to gender roles in productive activities.^63^^,^^64^ Gender roles often hinge on human subsistence strategies, which are shaped by ecological conditions and the demand for specialized skills.^49^^,^^65^ Indeed, sexual division of labor in resource exploitation, combined with economic motives, often dictates whether wealth is passed down through the paternal or maternal line.^2^^,^^20^^,^^66^ For instance, in societies where land is a crucial asset, the gender actively engaged in subsistence productive activities usually secures inheritance rights.^67^ Drawing on both ethnographic information and empirical data, we provide sufficient evidence that there are demographic changes in subsistence system from traditional, agriculture and pastoralism, to non-traditional productive economy with market-oriented production activities, aligning with theoretical models and findings from comparative cross-cultural studies.^2^^,^^17^^,^^30^^,^^45^^,^^48^^,^^66^ Agriculture, animal husbandry, and market economy have successively become the primary sources of income for local residents at different periods. The flexibility of gender roles in certain cultures allows inheritance system to evolve in response to changes in labor dynamics.^12^

By examining the primary livelihood activities of households, it is clear that gender preferences in inheritance system are subsistence-specific, establishing a connection between generational changes in gender bias within inheritance systems and demographic shifts in subsistence strategies. Initially, the primary source of household income was farming. Since 1960, the cultivated land area in this region had been continuously decreasing and remained below 30,000 mu since 1980 (15 mu = 1 ha).^62^ Our results indicate that women benefit more from inheriting family wealth when subsistence production activity is agriculture. Since women contribute more labors to agricultural activities,^62^ the decline in agriculture reduces their significances, which is likely to be reflected in gender-biased resource allocation. In settings where men primarily engage in fishing and women dominate in horticulture, such gender-based inheritance motivations are less pronounced in the absence of men.^68^ Ethnographic records suggest that local animal husbandry expanded rapidly after 1975 and has fluctuated within a certain range since 2000.^62^ In grazing activities, productivity is strongly related to the ability to defend animals, with men primarily responsible for protecting livestock.^65^ Previous researchers have reported that in communities where herding predominates and male labor is more valued, sons commonly obtain land and livestock inheritance.^69^^,^^70^^,^^71^ Our results also show that men are more likely to be an inheritor when grazing is the main source of livelihood. Therefore, the growth of pastoralism and the decline of agriculture both contribute to reduce gender gap in inheritance system. However, after 2000, non-traditional industries with more market integration developed rapidly and have become a significant source of income since 2010. Furthermore, our findings indicate no significant differences between men and women to become an inheritor when non-traditional production activities are the primary source of income, suggesting that non-traditional economy tends to erode gendered disparities. The rapid development of non-traditional economies with market integration, replacing animal husbandry as the primary source of local income, is likely to strengthen women’s status, preventing a complete shift toward a patrilineal inheritance system. Efforts to narrow the gender gap, such as initiatives to enhance women’s financial independence, have enabled them to make significant economic advances.^54^ In our study area, the post-2000 compulsory education policies have provided women with more equal opportunities in education and employment.^72^ Coupled with socio-economic advancements such as the expansion of lowland towns and improved infrastructures,^62^^,^^73^ there has been a noticeable shift in traditional gender roles, particularly in rural settings.^20^^,^^74^^,^^75^ The progressive reduction of gender bias in modernization, together with global educational progress,^76^ suggests a gradual softening of traditional norms regarding marriage and lineage, reflecting a broader trend toward gender equality. Similar results were found in a matrilineal group in southwestern China,^77^ where the kinship system was observed to transition from matrilineal to patrilineal. Although a patriarchal system has been more common throughout traditional Chinese history, an unbiased inheritance system is now emerging alongside a demographic transition.^78^^,^^79^

Additionally, our study investigates the associations between demographic changes in gender preferences within inheritance systems and demographic shifts in sibling composition and subsistence system, respectively. The results demonstrate that subsistence strategy, rather than sibling configuration, correlates strongly with gender-specific inheritance system. To what extent this is driven by societal norms from the wider Chinese economy or contact with those from outside the area (as in recent research from another location in the southwestern China^80^) is not clearly known.

Kinship systems, by defining roles, rights, and responsibilities within a society, are deeply intertwined with gender status and influence how different genders form social connections.^21^ Most studies suggest that men occupy more central positions in social interactions. For instance, study based on US Census, shows that men tend to create more extensive networks with a greater number of connections.^81^ The study based on Facebook profile pictures from nine world regions, indicated that men are anticipated to manage more extensive networks and establish social alliances.^26^ Recent research conducted in a patrilineal village shows that men who participate more in costly religious practices receive more nominations compared to women.^82^ Therefore, the elevated social roles typically held by men appear to be the key factor that enables them to secure a more advantageous position in social interactions. Cultural norms, social status, and factors like gender autonomy and power have long been intertwined with inheritance system.^16^^,^^18^ Previous research also posits that allocation of rights and responsibilities is coordinated with local residence rules and inheritance system.^13^ Gender-specific social status often dictates who holds authority within kinship networks and how resources are allocated. In many cultures, men’s higher status is reflected in their control over inheritance rights and property, while women’s access to these resources is limited by both legal and cultural barriers.^83^ Mattison et al. assessed gender-specific network centrality in two distinct villages, one matrilineal and one patrilineal, and their results indicate that gender dynamics within social networks are reshaped by local kinship norms that define gender roles.^21^ Here, we conduct economic games among a focal population in two separate periods and the results show that women received greater benefits from economic games in 2015, while there was no gender disparity in 2021. These results visually demonstrate the varying gender disparities in social interactions, consistent with the demographic transformation in gender-biased inheritance systems. We provide direct evidence that there has been a decline in women’s previously dominant status, not only in inheriting parental wealth but also in receiving village-level support. There has been limited attention to how gender differences in social interactions change as economies transition from traditional to market-oriented systems. Our study addresses this gap, revealing that as modern production integrates into traditional subsistence lifestyles due to market integration, substantial societal and cultural changes occur.^50^^,^^53^^,^^68^^,^^84^^,^^85^ This study suggests that kinship structures do reflect how gender social status is defined.^86^

### Conclusion

This study explores the evolving dynamics of gender bias, focusing on both within-family resource allocation and group-level interactions. We find that there are demographic shifts in gender-biased inheritance system that the strong female preference observed in older generations has diminished in recent generations, through analyzing extensive demographic data spanning over 70 years from 17 Tibetan villages. We consider two potential causes for this shift: demographic changes in sibling configuration and subsistence strategies. Our findings show that gender differences are consistent across sibling configurations. However, gender preference in inheriting family wealth is subsistence-dependent, with agriculture advantageous to women, pastoralism advantageous to men while non-traditional economy eroding gender bias. Additionally, there are demographic shifts in subsistence strategies from agriculture to more market-originated economy. Therefore, demographic shifts in subsistence system relate to the changes in gender bias within inheritance practices from matrilineal toward non-unilineal. In addition, results from economic game experiments conducted during two survey periods (2015 and 2021) show that the strong inclination to invest in females seen in 2015 had disappeared by 2021. Our findings clearly demonstrate a demographic shift in gender preference, moving from a female bias toward a more neutral stance, both in familial resources distribution and broader social interactions. Our research indicates that it is crucial to consider subsistence strategy and a wide range of socio-ecological factors when studying shifts in sex preferences and kinship systems.

### Limitations of the study

This study has certain limitations. Although our demographic data spanning over 70 years are valuable, the period during which market economy has become the primary source of income is relatively short. Based on the current results, we can only infer a connection between market economy and an unbiased inheritance system. However, there is also the possibility that the region is currently in a transitional phase from a matrilineal to a patrilineal inheritance system. Additionally, our economic games focus on whether interviewees prefer men or women to gain benefits, without the expense of their own interests or providing them benefits. Type of social interaction is a crucial factor. Therefore, further research is necessary to explore gender bias across different forms of social interactions and various inheritance systems.

## Resource availability

### Lead contact

For further information and resource requests, please direct your requests to the lead contact, Juan Du (dujuan@lzu.edu.cn).

### Materials availability

This study did not produce any novel reagents or materials.

### Data and code availability

• The data used to produce the results in the manuscript and supplementary materials presented, tables, or figures are available on GitHub and is publicly available as of the date of publication. The DOI is listed in the key resources table.
• All original code has been deposited on GitHub and is publicly available as of the date of publication. The DOI is listed in the key resources table.
• Any additional information required to reanalyze the data reported in this paper is available from the lead contact upon request (dujuan@lzu.edu.cn).

## Acknowledgments

This study was supported by 10.13039/501100001809NSFC (no. 32401289). J.D. and R.M. were also supported by European Research Council Advanced Grant (grant number: 834597). We would like to thank local people who took part in this study by answering our questionnaires. Many thanks to local assistants Luo Ti, Zhaxiqidan, and their family members who provided lots of help and support for us when collecting data.

## Author contributions

Conceptualization, J.D. and R.M.; methodology, J.D. and Y.H.; investigation, J.D., Y.H., P.B., and L.Z.; data curation, Y.H.; formal analysis, Y.H.; visualization, Y.H.; supervision, J.D. and R.M.; funding acquisition, J.D. and R.M.; project administration, J.D. and R.M.; writing – original draft, Y.H.; writing – review & editing, J.D., Y.H., and R.M.

## Declaration of interests

The authors declare no competing interests.

## STAR★Methods

### Key resources table

**Table:** 

REAGENT or RESOURCE	SOURCE	IDENTIFIER
**Deposited data**		

Data used to produce the results in the manuscript and supplementary materials presented	This paper	Database: https://github.com/9YamingHuang42/Inheritance-system-and-gender-preference-gifts-giving

**Software and algorithms**		

Code for all models and analyses	This paper	GitHub: https://github.com/9YamingHuang42/Inheritance-system-and-gender-preference-gifts-giving

### Experimental model and study participant details

#### Institutional permission

Lanzhou University granted ethical approval for the study (reference EAF2023001). Informed consent was secured from both the local government and all individual participants.

#### Study population

Data collection is conducted in a town located in the northwest region of Diqing Tibetan Autonomous Prefecture, Yunnan province, China. This administrative town encompasses three central townships.^87^ The majority of the local population are Tibetans, accounting for over 90% of residents who share a common ethnic background and language.^88^ Educational levels in the area are generally limited, with formal schooling becoming prevalent only after the compulsory education policies were introduced in 2000.^72^

#### Ethnographic settings

Our study site is one of the three townships, including 17 villages. In our research region, the prevailing inheritance system is order-biased primogeniture, which favours the eldest children regardless of gender, providing them with the greatest share of parental resources, including Tibetan houses, agricultural land, and support for child-rearing (see electronic Figure S1).^89^^,^^90^ Later-born children often marry individuals from the same village or township.^62^^,^^91^ Consequently, most residents remain in their hometown or close-knit community throughout their lives,^62^^,^^89^ with marriages predominantly occurring at the village level.^90^ Due to the implementation of China planning policies in 1980s, the number of children per couple was limited, significantly altering reproductive behaviours.^62^^,^^90^ By the early 21st century, reproductive behaviours of local residents had changed, with most couples having one or two offspring. The shifts in reproductive behaviours directly resulted in a reduction in the number of siblings (details seen in electronic supplementary materials Methods S1 section). Ethnographic publications reported that subsistence strategy in our study region is a mix of farming and herding.^62^ Farmland, which once provided the primary source of income, is now typically leased to companies, with local residents employed for agricultural work. The grazing method is extensive, primarily involving the raising of yaks and plateau yellow cattle. In 2007, the local economy was predominantly based on animal husbandry, supplemented by agriculture.^62^ However, with the integration of market economy, tourism (such as running shops and working as tour guides) and transportation industries (such as operating excavators and long-distance transportation) have rapidly developed, leading to a decline of pastoralism and agriculture (details seen in electronic supplementary materials Methods S1 section). There is also a clear gender division of labour. Women played a crucial role in farming activities.^62^ As economy developed, men predominantly entered the marketplace, particularly in construction and transportation sectors.^62^ Recently, an increasing number of women has been engaging in part-time employment, for example, working for companies.^91^

Our research area covers all the villages of one township, totalling 17 villages (see Table S1). Although these 17 villages are geographically close, there are some differences in accessibility of natural resources and distance to markets. Four villages (villages 14-17) locate in mountains, farthest from markets. Eight villages (villages 1-8) are closest to markets, thus residents have the highest degree of market integrations. The rest five villages (villages 9-13) are in transition stage between these two aforementioned regions. To reach markets, residents need to travel over one mountain, with the degree of market economy involvement decreasing progressively from outer to inner regions. More details about differences in accessibility of natural resources and distance to markets among 17 villages are in electronic supplementary materials Methods S1 section.

### Method details

#### Data collection of inheritance system

Demographic data was gathered in 2015 and 2021 from a township encompassing 17 Tibetan villages. The work was carried out by JD, YMH, LQZ, and PPB, with the assistance of local interpreters. We conducted comprehensive interviews with every adult in each household, using paper questionnaires to collect both individual and household demographic data. The survey included details such as name, age, Zodiac sign (for more accurate birth year determination), gender, educational attainment, birthplace at the village level, sibship details (both alive and deceased, their names, ages, Zodiac sign, birth order, inheritance, current residence location, if having the same father and if having the same mother), and parental details (name, age, Zodiac sign, current residence location, and if deceased, the years and location of death). In 2015, our study covered 13 villages (villages 1-13) and the four villages in mountains (villages 14-17) were omitted. In 2021, these four villages were included and demographic data of 13 villages that had been collected in 2015 were updated. We obtain detailed information from 677 households, covering 3836 local individuals, including those who lived in our study area in 2015 but had moved away or passed away by 2021. In 2021, there were 3586 local residents living in our study site (1791 males and 1795 females) (see Table S1). Adult respondents retrospectively reported the main livelihood strategies of their families when they were unmarried. More details about data collection and management are in electronic supplementary materials (Methods S1).

#### Data collection of gifts received games

Gift games were conducted in both 2015 and 2021. Each adult participant received 15 yuan, which they could distribute as gifts to other married adults from different households within the study area. All participants were required to distribute their entire amount to at least one but up to three other persons and were prohibited from gifting to any household member or retaining any money for themselves. In 2015, there were 188 participants (148 recipients and 225 gifts), while in 2021, there were 73 participants (103 recipients and 125 gifts) (see Table S3 in electronic supplementary materials).

### Quantification and statistical analysis

#### Models about inheritance system

The analyses included individuals born at the study site with complete sibling records. Individuals who moved to other townships or had passed away are included, providing insights into inheritance system of older generations. Birth cohorts are divided into six groups. Sibling configuration is categorised into three groups: equal number, more sisters and more brothers. Retrospective subsistence strategy is divided into four groups: agriculture, semi-agriculture and semi-pastoralism, pastoralism and non-traditional industry. The non-traditional industry includes all types other than the first three, including running a business, having a stable job, building houses, operating excavators, and having a part-time job, etc. The study ultimately compiles 742 complete sibling datasets (i.e., from same mother ID), including 2234 individuals and 766 inheritors (see Table S4). Annual ethnographic data are sourced from the China Yearbook Database (https://kns.cnki.net/kns/advsearch?dbcode=CYFD) and local historical records 62.

This research employs generalised linear mixed models (binomial)^92^^,^^93^ to analyse the impacts of gender on the probability of inheriting family wealth defined as remaining at and taking over the natal home, with being an inheritor as the response variable (response variable details seen electronic supplementary materials Methods S1 section), Mother ID is used as a random effect. Cohorts, sibling configuration and subsistence strategy are included in models. A directed acyclic graph (DAG) is used to identify the cause-effect relationships intended for modelling (details seen in electronic supplementary materials Methods S1 section and Figure S2A).^94^ We build model 10 to test sex-biased demographic changes in inheritance system and add different interactions in order to explore possible drivers, building sibling-configuration-specific gender bias (model 11), subsistence-specific gender bias (model 12), cohort-specific and sibling-configuration-specific gender bias (model 13), cohort-specific and subsistence-specific gender bias (model 14) and all these three interactions, respectively (see Table S2 in electronic supplementary materials). Equations 1, 2, and 3 are common equations used in all models for inheritance system. Let *Y*_ij_ as represents the binary outcome for the *i*-th observation within the *j*-th mother ID. The probability of being an inheritor (e.g., event occurrence) is *p*_ii_. A logit link function is commonly used for binomial outcomes, relating the probability to the linear predictor. β0 represents the intercept, βk (k = 1, 2, … , N) represents coefficient for predictor Xk_ij_, Xk (k = 1, 2, … , N) represents *k*-th predictor, α1 represents coefficient for the interaction between X1 and X2, and b_j_ represents random effect for the *j*-th mother ID, assumed to follow a normal distribution. Note that main and interaction effects are represented mathematically in Equation 3. The specific variables and interaction effects included are detailed in Table S2. The comprehensive results are detailed in Table S5. For straightforward interpretation, this study primarily relies on models 10, 11, and 12, with models involving multiple interactions (models 13, 14, and 15) serving as supplementary analyses. Interactions are exhibited by comparing gender differences when fixing another variables, as detailed in Tables S6–S8 and Figures 1 and 2A–2C.

(Equation 1) $$p_{ij}=\mathrm{Pr}\left(Y_{ij}=1\right)$$

(Equation 2) $$\log\;it\left(p_{ij}\right)=\log\left(\frac{p_{ij}}{1-p_{ij}}\right)$$

(Equation 3) $$\log\;it\left(p_{ij}\right)=\beta 0+\beta 1\times X1_{ij}+\beta 2\times X2_{ij}+\ldots +\beta N\times XN_{ij}+a1\times \left(X1_{ij}\times X2_{ij}\right)+b_{j}$$

All statistical analyses are conducted in R (v. 4.2.3)^95^ using the packages dagitty,^96^ glmmTMB,^97^ emmeans,^98^ plyr,^99^ dplyr,^100^ and MuMIn.^101^ Plots are generated using ggplot2,^102^ dotwhisker^103^ and ggalt.^104^ We used an alpha level of 0.05 for all statistical tests.

#### Models about gift received games

Participants, termed "egos", have the capacity to give gifts, whereas "alters" are the potential recipients. Consequently, dyadic relationships are established between each participant and each his/her potential candidate. This setup creates 417,263 potential dyadic relationships for 188 participants in study-1 (2015) and 169281 potential dyadic relationships for 73 participants in study-2 (2021) (see Tables S3 and S9 in electronic supplementary materials). For each dyad, if the ego nominates the alter as a recipient, the response variable is the monetary amount received. Conversely, if no nomination occurred, the response is recorded as zero. Ages are standardised. Regarding post-marital dispersal patterns, we record whether individuals stay in their natal village after marriage, resulting in two groups: philopatry (i.e., staying in the natal village) and dispersal (i.e., residing outside the natal village) (details seen in electronic supplementary materials Methods S1 section).

Using generalised estimating equations (Poisson), we assess the influences of gender on participants' preference in gifts games. By comparing the effects of gender between study-1 (2015) and study-2 (2021), we examine whether there are any differences between two survey times. The generalised estimating equation (GEE) approach is based on the quasi-likelihood theory accommodating correlated data without presuming a specific distribution of responses.^105^^,^^106^ Equation 4 are common equations used in all models for gifts received. μi as represents the expected count for the i-th observation. β0 is the intercept, βk (k = 1, 2, … , N) represents coefficient for predictor Xki, Xk (k = 1, 2, … , N) represents k-th predictor, and α1 represents coefficient for the interaction between X1 and X2. Equation 4 mathematically represents both the main effects and interaction effects of the variables under study. For a detailed breakdown of these variables and their specific interactions, please refer to Table S2. Our analysis use an exchangeable correlation matrix within the GEE frameworks. A DAG is also employed to identify the cause-effect relationships (details seen in electronic supplementary materials Methods S1 section and Figure S2B) and get the final model based on a set of candidate models using the quasi-likelihood under the independence model criterion (QIC) (see Table S2 in electronic supplementary materials).^107^ The full model is presented in Table 1. Interactions are exhibited by comparing gender differences when fixing another variables, as detailed in Table S10 and Figure 4.

(Equation 4) $$\log\left(\mu_{i}\right)=\beta 0+\beta 1\times X1_{i}+\beta 2\times X2_{i}+\ldots +\beta N\times XN_{i}+a1\times \left(X1_{i}\times X2_{i}\right)$$

All statistical analyses are conducted in R (v. 4.2.3)^95^ using the packages dagitty,^96^ geepack,^108^ and MuMIn.^101^ Plots are generated using ggplot2^102^ and ggalt.^104^ We used an alpha level of 0.05 for all statistical tests.
