# Supplementary material for: A rapid decline in gender bias relates to changes in subsistence practices over demographic changes in a formerly matrilineal community
Source: iScience. 2025 Jan 31;28(2):111926. doi: 10.1016/j.isci.2025.111926 (PMC11872634; doi:10.1016/j.isci.2025.111926)
Supplement: Document S1. Figures S1–S4, Tables S1–S12, and Methods S1 [file mmc1.pdf]

iScience, Volume *28*

Supplemental information

A rapid decline in gender bias relates to changes in subsistence practices over demographic changes in a formerly matrilineal community

Yaming Huang, Pengpeng Bai, Liqiong Zhou, Ruth Mace, and Juan Du

This PDF file includes:

Methods S1: More details about study settings, data collection, data management, variables and DAGs, related to the STAR Methods

Figure S1: Self-reported main category of each household's expenses.

Figure S2: Directed acyclic graphs.

Figure S3: Proportion of each economic sources across years in administrative town.

Figure S4: Proportion of household income by economic types and study time.

Tables S1: Descriptive data of individuals residing in 17 Tibetan villages in 2021.

Tables S2: Candidate set of models.

Tables S3: Descriptive statistics of gift**s** giving game.

Tables S4: Descriptive data for variables using in inheritance models.

Tables S5: Estimates in inheritance models predicting the probability of inheriting family wealth.

Tables S6: Compare sex difference when fixing the cohorts from Model 10, 13, 14 and 15.

Tables S7: Compare sex difference when fixing sibling configuration from Model 11, 13 and 15.

Tables S8: Compare sex difference when fixing subsistence system from Model 12, 14 and 15.

Tables S9: Descriptive data for variables using in gifts received models.

Tables S10: Compare sex difference when fixing study time from gifts received models.

Tables S11: Proportion of parents' main livelihood form (when interviewees were unmarried), related to figure 3B.

Tables S12: Proportion of income from agriculture, pastoralism, and non-traditional industry across years, related to figure S3.

Methods S1

Reproductive behaviours controlled by policies

The family planning policy had advocated in our study site since 1979. In 1981, relevant rules were issued stipulating that urban residents could not have more than two children, while rural residents could not have more than three. The two-child policy was implemented in 1991, regardless of whether the residents were in rural or urban areas, with a encouragement to have only one child. By the early 21st century, reproductive behaviours of local residents had changed, with most families having one to two children. The shifts in reproductive behaviors directly resulted in a reduction in the number of siblings.

Subsistence strategies

Ethnographic literatures reported that subsistence in our study region is a mix of farming and herding. In agriculture, the locals mainly grow barley, potatoes, maca and turnips. The agricultural production activities of the local residents still remain at a relatively traditional level, mostly using traditional farming tools such as hoes and rakes. In 2005, the local government began to introduce modern farming techniques and in 2013, farmers began to cooperate with companies, generating a producing mode called ‘company + coops + farmer’. Local residents generally lease their land to companies and then are hired by companies for agricultural labors. As a result, although the residents still engage in various agricultural production activities, source of income has shifted from a traditional agricultural economy to a market-integrated economy, primarily based on land rent and wages from part-time jobs. The grazing method is extensive, primarily involving the raising of yaks and plateau yellow cattle. The pastures are divided into summer and winter pastures, with rotational grazing practiced. Grazing areas are divided and belong to different villages. In 2007, the local economy was predominantly based on animal husbandry, supplemented by agriculture, with livestock farming accounting for 62.9% of the total income. However, with the integration of market economy, tourism (such as running shops and working as tour guides) and transportation industries (such as operating excavators and long-distance transportation) have rapidly developed, leading to a decline in the proportion of pastoralism and agriculture. Currently, the number of livestock raised by households varies greatly: most families raise livestock primarily for self-sufficiency, while families that engage in grazing for income purposes usually conduct a large-scale grazing. There is also a clear gender division of labour. Women played a crucial role in farming activities. As economy developed, men predominantly entered the marketplace,

particularly in construction and transportation sectors. Recently, an increasing number of women has been engaging in part-time employment, for example, working for farming companies.

Differences in accessibility of natural resources and distance to markets among 17 villages

Our research area covers all the villages of one township, totaling 17 villages. Although these 17 villages are geographically close, there are some differences in accessibility of natural resources and distance to markets. 17 villages can be roughly divided into three major regions. Villages 14-17 are located in mountains, making residents easier to engage in grazing activities and gathering mushrooms. These four villages are farthest from markets and residents have to travel over the mountains to get in and out, thus residents need most time to reach markets. However, there are some market economies involvement due to land being leased to companies and then residents being hired. In recent years, sand quarries in the nearby forests of village 14 has driven the residents engaging in transportation activities (i.e. transporting sand). Villages 1-8 are closest to markets, thus residents have the highest degree of market integrations. Villages 3 and 4 are primarily engaged in non-traditional economic activities and having most residents with stable jobs among all the 17 villages. The last five villages, villages 9-13, are in the transition stage between the two aforementioned regions. To reach markets, residents need to travel over one mountain. Village 9 has more residents engaged in market economy activities, while village 13, located in the innermost part of these five villages, has a higher proportion of residents involved in grazing.

Details about data collection

Data collection consisted of two main parts: household and individual information. Initially, we gathered demographic data for each household member, such as name, age, birth year, Zodiac sign (for precise birth year calculation), gender, detailed marital status, relationship to the head of the household, educational attainment, birthplace at the village level, and information about their parents (including name, age, birth year, Zodiac sign, current residence, and if deceased, the year and location of death). Following this, we collected data on household assets, income and expenses. For family assets, we documented the number of cattle, pigs, sheep, and land (including details on how much was leased versus farmed independently and the specific crops grown). Family income data included detailed sources such as income from livestock, milk products, fungi, death subsidies

for animals, housing subsidies, other subsidies, part-time jobs, wages, tourism revenue, and other sources. We also collected comprehensive data on main household expenses.

Detailed marriage information was recorded, including the spouse's name, marriage age, duration, year of divorce, current residence, and the number of living and deceased offspring. Similarly, we documented sibling information, covering birth order, name, age, Zodiac sign, current residence, inheritance and if deceased, the year and location of death, as well as whether they share the same father or mother. Detailed offspring information was also collected, with a focus on name, age, Zodiac sign, current residence, and if deceased, the year and location of death, along with the names of the father or mother. Spousal information was linked with offspring data regarding the father or mother.

We also traced historical livelihood activities to gather insights into subsistence systems related to inheritance practices, asking questions like "What was the main source of family income when you were unmarried?" It's important to note that our data were collected using paper questionnaires. Although the electronic version offers the convenience of skip logic, which automatically advances through questions, it may omit additional information that respondents might inadvertently provide. Moreover, the use of paper questionnaires helps build trust with interviewees in rural areas.

Details about data management

For each individual, we use the coding method of "region number + personal number" to generate their unique identity number. Each household is also coded with a unique household number. First, we code person ID and then confirm mother ID and father ID using information such as the parents' names, whether they are alive, current residence, age, etc. The mother ID and father ID are further verified using sibling information and reproductive details. By coding the mother ID and father ID, we can link siblings together. Note that we do not use a parent ID to connect sibling information; instead, we separately code father ID and mother ID. Separately coding mother and father ID helps us distinguish among full siblings, paternal half-siblings, and maternal half-siblings. The survey conducted in 2021 was based on data collected in 2015. In 2021, we updated family members for each household and interviewed households that were not interviewed in 2015. The updated information are included in a column explaining why this individual is not in this family in 2021 and their current whereabouts. For example, if a person was not in her or his previous household because he or she got married and joined spouse’s family, marriage year and

current residence would been shown in this column and a new row about this person with the same person ID would been added in the household where he or she moved to.

Practice of zuò jiā (坐家)

In our study site, the Tibetans engage in the practice of zuò jiā (坐家) that parents will choose one offspring who will co-reside with his or her parents after marriage and inherit the most part of parental wealth. Because the decision of choosing which offspring is commonly made before any offspring get married, there are unmarried individuals in our models. The philopatric offspring should co-reside with their parents after marriage, while others will disperse from natal family after they get married. In common, dispersing individuals are only allowed to take their own luggage away from their natal family. Therefore, the philopatric individuals inherit the most of parental wealth, including valuable Tibetan houses, farmland and livestock which are most important part of family economy.

The control variable about dispersal pattern in gifts received models

We note that the definition of dispersal pattern is different from the one used in models of inheritance system. In inheritance system models, dispersal pattern is determined by the traditional practice of zuò jiā (坐家) in our study area. One offspring will be chosen to zuò jiā (坐家), who will stay at their natal family after marriage and inherit the most of parental wealth. However, in gifts received models, dispersal pattern is defined by whether staying in their natal community. When playing economic games, broader social interactions is more important. Staying in natal community means having more acquaintances and relatives, so we define dispersal pattern in a broader level but not in family level.

Directed acyclic graphs

The directed acyclic graph (DAG) aims to identify causal factors that impact both the outcome and exposure simultaneously, thereby closing backdoor paths. Additionally, in order to identify direct causal effect from indirect causal effects, the DAG further focuses on causal factors of the exposure’s descendants.

For inheritance system, inheritance is outcome and gender is exposure. Gender and birth order are assumed to influence the probability of being an inheritor, based on the matrilineal structure and order-biased primogeniture. Because of family planning policy implementation in 1980s, cohorts

are expected to influence inheritance, birth order, sibling configuration. As an increasing number of local people enters the marketplace and are employed, the DAG assumes the effects of cohorts on subsistence. Parents prefer children who make main contribution to livestock activities, therefore, subsistence is expected to sharp inheritance. When sibling configuration changes, meaning competition intensity among siblings changing, the DAG expects that sibling configuration cause the changes in inheritance system. Although there are not open biasing paths, interactions of gender with three other factors - cohorts, sibling configuration and subsistence - are added into models.

For economic games, the amount of gifts received is outcome and alter gender is exposure. Firstly, gender, education, dispersal pattern, economy and age of egos and alters are expected to influence the distributions of gifts. Gender, education, dispersal pattern, economy and age of alters are assumed to have effects on individual social status and benefits from social interactions. Survey period is also expected to have influences on the outcome. For alter gender, which the DAG focuses on, survey period and ego's age and gender are assumed to influence the choices of recipients' gender. Hence, these three factors should be added into models as they are confounders that could introduce backdoor paths. Alter gender is expected to influence alter dispersal pattern which open the path enable alter gender to indirectly transmit information to outcome. In order to close indirect causal paths, alter dispersal pattern and its confounders (e.g., factors impact both the outcome and exposure simultaneously) should be considered. The DAG expects dispersal pattern of ego and age of alters influence dispersal pattern of alter, so these two factors should be included into models. In conclusion, survey period and ego's age and gender should be included into models for controlling confounders, and alter dispersal pattern, ego dispersal pattern and alter age should be added into models for controlling indirect casual paths.

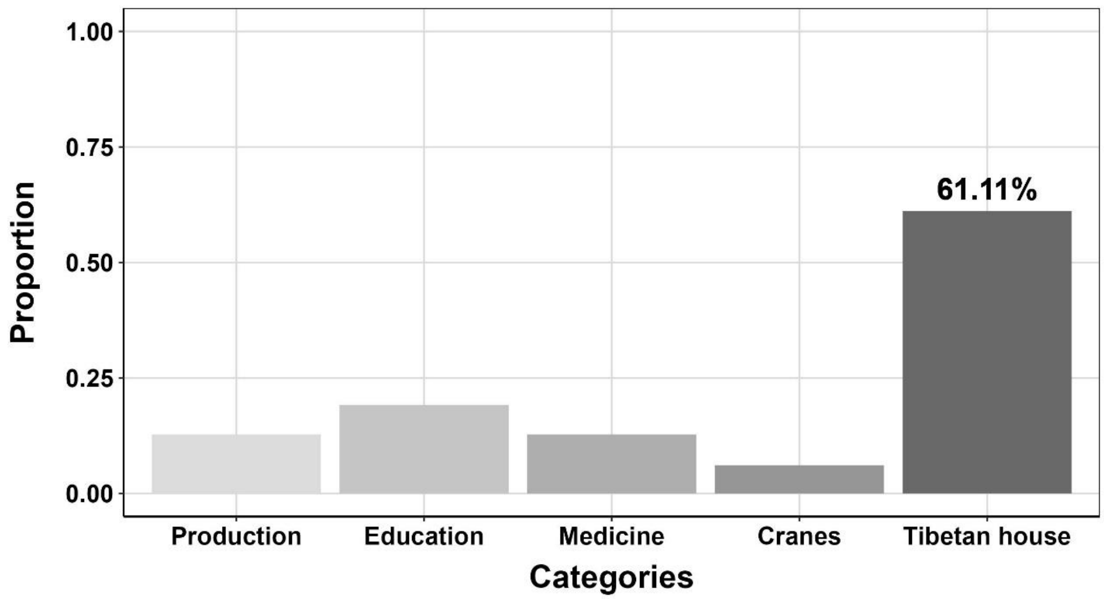

Figure S1: Self-reported main category of each household's expenses. N = 360 household economic information is used in this plot, excluding households regarding the daily expense as the only main expense. Production represents expense in productive activities, such as buying agricultural chemicals, animal feed, etc. Cranes represents expense of buying cranes. Tibetan house represents expense in house, like preparing materials, construction, reconstruction and so on.

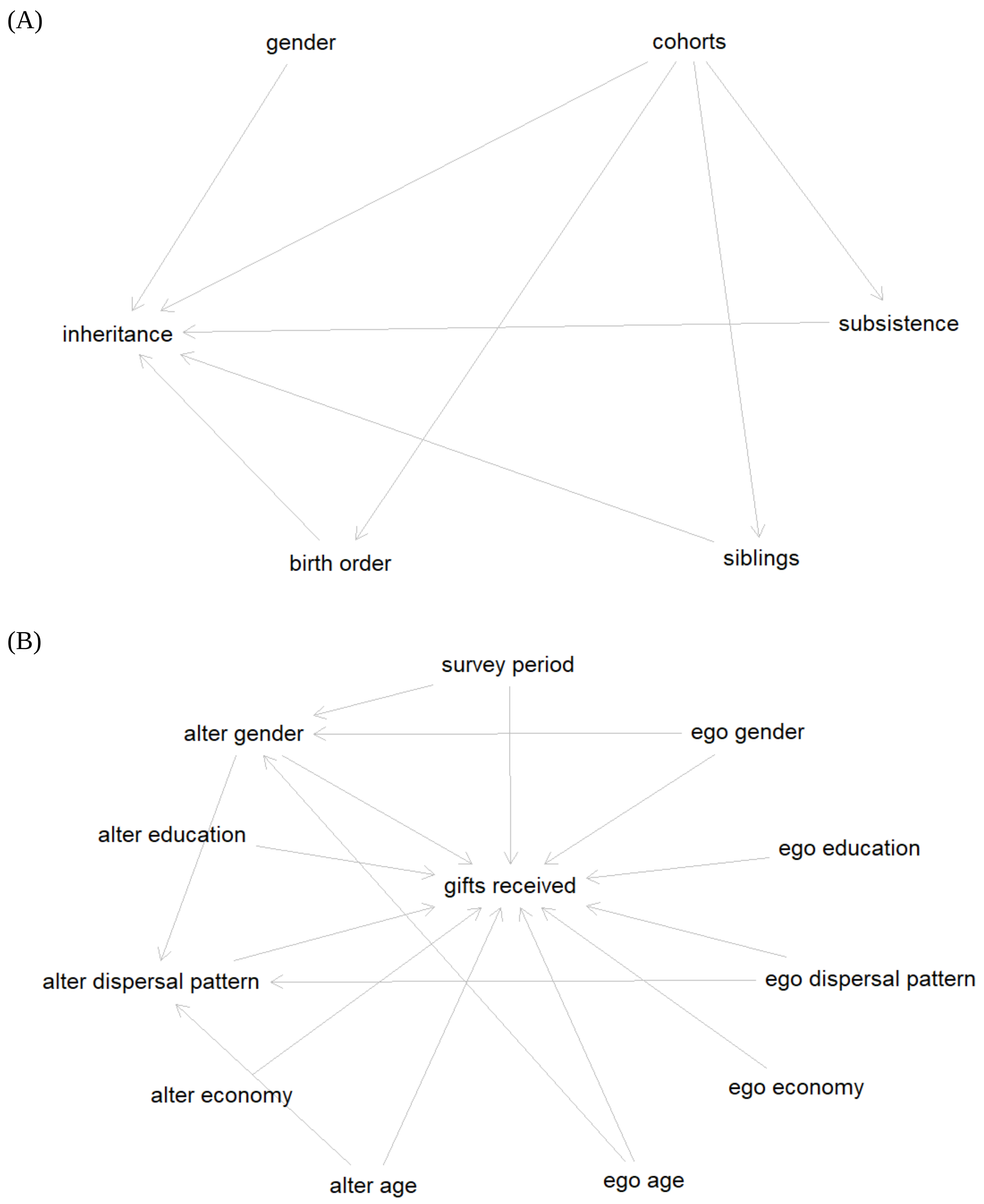

Figure S2: Directed acyclic graphs. (A) Gender is the exposure and inheriting family wealth is the outcome. (B) Alter's gender is the exposures and gifts received is the outcome.

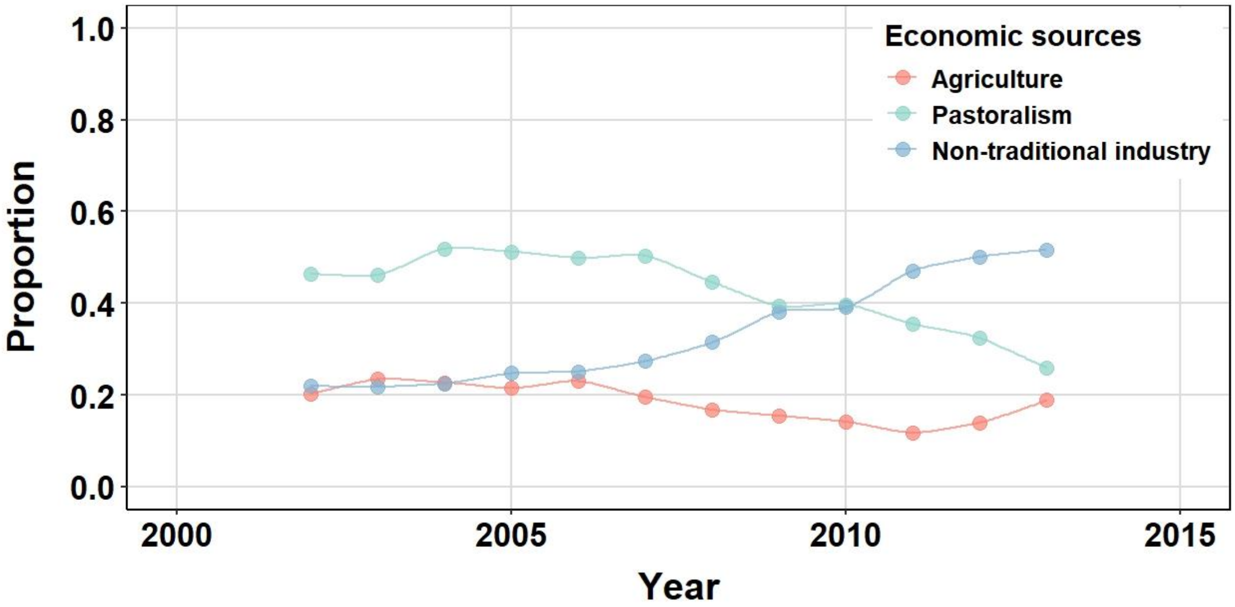

Figure S3: Proportion of each economic sources across years in administrative town. Our study site is one of the three central townships in this administrative town. Red point and line represent proportion of income from agriculture. Blue point and line represent proportion of income from pastoralism. blue point and line represent proportion of income from non-traditional industries, including income from services, transportation, construction and so on. Exact proportions are given in Tables S11.

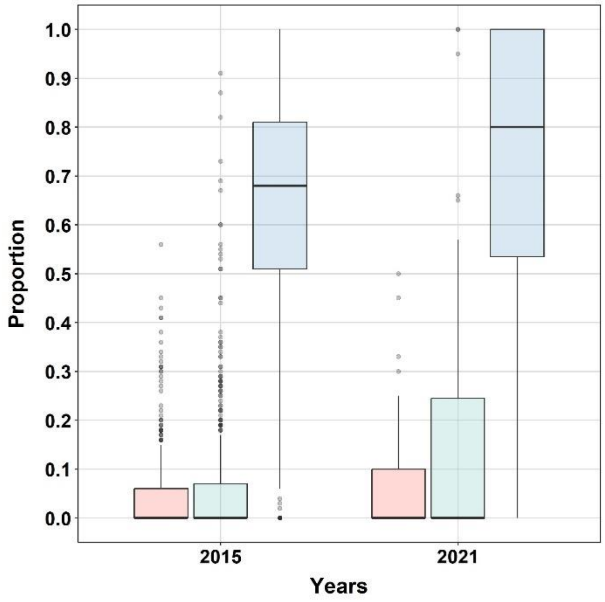

Figure S4: Proportion of household income by economic types and study time. Red represents agriculture green represents pastoralism and blue represents non-traditional industries (see also table S10).

Table S1: Descriptive data of individuals residing in 17 Tibetan villages in 2021

Village_ID	Number of household	Population	Females / Males	Mean age (min, max)
V1	108	523	266	35.70 (1, 90)
			257	
V2	48	241	121	36.59 (1, 82)
			120	
V3	51	255	117	37.01 (2, 88)
			138	
V4	26	139	72	33.76 (1, 85)
			67	
V5	11	45	23	39.84 (2, 87)
			22	
V6	28	157	81	35.76 (1, 84)
			76	
V7	45	237	122	36.54 (1, 86)
			115	
V8	57	308	158	36.28 (1, 86)
			150	
V9	66	337	172	36.93 (0, 89)
			165	
V10	69	373	192	35.10 (0, 91)
			181	
V11	36	202	106	35.53 (0, 87)
			96	
V12	34	211	105	35.57 (0, 91)
			106	
V13	27	165	78	35.70 (1, 95)
			87	
V14	24	127	64	37.06 (0, 78)
			63	
V15	20	104	43	36.83 (1, 93)
			61	
V16	16	96	44	36.82 (1, 84)
			52	
V17	11	66	31	34.73 (4, 76)
			35	

Table S2: Candidate set of models. Models for inheritance predict the probability of inheriting family wealth and gifts received models predict the amount of money received in dyads (ego–alter pairs) within the same township.

Model	AIC /QIC	ΔAIC/Δ QIC	Weight
**Inheritance**			
15: cohort + gender + sibling configuration + subsistence + cohort : gender + sibling configuration : gender + subsistence : gender	2753.91	3.31	0.138
**14: cohort + gender + sibling configuration + subsistence + cohort : gender + subsistence : gender**	**2750.60**	**0.00**	**0.722**
13: cohort + gender + sibling configuration + subsistence + cohort : gender + sibling configuration : gender	2767.53	16.94	0.000
12: cohort + gender + sibling configuration + subsistence + subsistence : gender	2753.88	3.29	0.140
11: cohort + gender + sibling configuration + subsistence + sibling configuration : gender	2775.83	25.24	0.000
10: cohort + gender + sibling configuration + subsistence + cohort : gender	2763.77	13.17	0.001
9: cohort + gender + sibling configuration + subsistence	2772.27	21.67	0.000
8: cohort + gender + subsistence	2776.48	25.88	0.000
7: cohort + gender + sibling configuration	2774.30	23.70	0.000
6: cohort + gender	2779.97	29.37	0.000
5: subsistence	2841.86	91.26	0.000
4: sibling configuration	2859.68	109.08	0.000
3: gender	2855.37	104.78	0.000
2: cohort	2802.89	52.30	0.000
1: intercept-only	2876.61	126.01	0.000
**Gifts received**			
**10: ego's age + ego's dispersal pattern + ego's gender + alter age + alter dispersal pattern + time × alter gender**	42821.78	0.00	1.00
9: ego's age + ego's dispersal pattern + ego's gender + alter age + alter dispersal pattern + time **+** alter gender	42862.40	40.62	0.00
8: alter gender	43260.92	439.13	0.00
7: time	43553.54	731.76	0.00
6: alter dispersal pattern	43320.44	498.65	
5: alter age	43327.02	505.24	0.00
4: ego's gender	43559.76	737.98	0.00
3: ego's dispersal pattern	43559.71	737.93	0.00
2: ego's age	43559.72	737.94	0.00
1: intercept-only	43557.81	736.02	0.00

The best-fitting models are in bold and analyzed in the main text

Table S3: Descriptive statistics of gifts giving game

Variable	Study-1 (2015)	Study-2 (2021)	X^2 (*P*)
No. participant (person)	188	73	/
No. recipient (person)	148	103	/
No. gift (number)	225	125	/
Mean (SD) $_{recipient-gift}$	1.52 (2.61)	1.21 (0.70)	/
No. money (yuan)	2605	964	/
No. disposable money (yuan)	2820	1095	/
Pro. money being used (yuan)	92.38%	88.04%	0.81 (0.37)
Mean (SD)$_{recipient-money}$	17.60 (37.57)	9.36 (8.57)	/
No. recipient candidate (person)	2223	2323	/
No. dyad (tie)	417263	169281	/

Recipient-gift represents the number of gifts that are received by recipients. Recipient-money represents the total amount of money that are received by recipients. As each participant is allowed to give gifts of 15 yuan, the total money could be used equaled to the number of participants times 15. Note that in each interviewing year, there are some participants that don’t use all 15 yuan, so we show the proportion of money being used in this table. Recipient candidate represents the people that participants are able to give gifts to, and they are the number of adult individuals in our study site in two interviewing years. And the dyads are obtained after deleting dyads that ego and alter belong to a same household

Table S4: Descriptive data for variables using in inheritance models.

Variables		*N* = 2234 (sibship = 742) N (%)	Range	Variable description
Sibling configuration	Equal number	443 (19.83)		Sibling configuration, comparing the number of sisters with the number of brothers, is divided into three groups: equal number, more sisters and more brothers. Note that individuals, who have no sibling, are categorize into the group named "equal number"
	More sisters	936 (41.90)		
	More brothers	855 (38.27)		
Subsistence system	Agriculture	863 (38.63)		The major household subsistence activity, through tracking back by asking "when you were unmarried, especially as a teenager, what was your family's or parents' main livelihood activity?", is divided into four groups
	Semi- agriculture and semi-pastoralism	887 (39.70)		
	Pastoralism	129 (5.77)		
	Non-traditional industry	355 (15.89)		
Gender	Female	1161 (51.97)		Gender
	Male	1073 (48.03)		
Cohort	<=1955	324 (14.50)	(1922, 2007)	The cohorts that are represented by individuals' birth years, are divided into six categories
	1956-1965	267 (11.95)		
	1966-1975	530 (23.72)		
	1976-1985	474 (21.22)		
	1986-1995	434 (19.43)		
	>1995	205 (9.18)		
Mother ID		N = 742		There are 742 sibships and we used the same mother ID to link the siblings

Table S5: Estimates in inheritance models predicting the probability of inheriting family wealth.

Variable	Model 9	Model 10	Model 11	Model 12	Model 13	Model 14	Model 15
Fixed effects							
Intercept	**0.38 [0.27 – 0.54] *****	**0.46 [0.32 – 0.68] *****	**0.39 [0.27 – 0.58] *****	**0.44 [0.31 – 0.62] *****	**0.47 [0.30 – 0.70] *****	**0.51 [0.34 – 0.75] *****	**0.50 [0.33 – 0.78] ****
Cohort: 1956-1965	1.05 [0.73 – 1.50]	0.97 [0.61 – 1.54]	1.05 [0.73 –1.50]	1.02 [0.71 – 1.47]	0.97 [0.61 – 1.54]	0.96 [0.60 – 1.52]	0.96 [0.60 – 1.52]
Cohort: 1966-1975	0.94 [0.68 – 1.28]	0.87 [0.58 – 1.30]	0.93 [0.68 – 1.27]	0.94 [0.69 – 1.29]	0.87 [0.58 – 1.30]	0.90 [0.60 – 1.34]	0.90 [0.60 – 1.34]
Cohort: 1976-1985	1.07 [0.78 – 1.47]	0.84 [0.55 -1.28]	1.06 [0.77 –1.46]	1.06 [0.77 – 1.46]	0.84 [0.55 – 1.28]	0.87 [0.57 – 1.33]	0.87 [0.57 – 1.32]
Cohort: 1986-1995	**1.97 [1.43 – 2.72] *****	1.45 [0.96 – 2.20]	**1.96 [1.42 -2.70] *****	**1.94 [1.40 – 2.68] *****	1.45 [0.95 – 2.20]	1.48 [0.97 – 2.26]	1.48 [0.97 – 2.26]
Cohort: >1995	**2.35 [1.57 – 3.50] *****	1.30 [0.78 – 2.17]	**2.34 [1.57 – 3.49] *****	**2.32 [1.55 – 3.47] *****	1.30 [0.78 – 2.16]	1.34 [0.79 – 2.28]	1.34 [0.79 – 2.28]
Male	**0.62 [0.52 – 0.75] *****	**0.38 [0.23 – 0.64] *****	**0.58 [0.38 – 0.90] ***	**0.39 [0.29 – 0.54] *****	**0.40 [0.21 – 0.78] ****	**0.28 [0.16 – 0.48] *****	**0.28 [0.14 – 0.57] *****
More sisters	1.17 [0.90 – 1.52]	1.16 [0.90 – 1.51]	1.10 [0.77 – 1.56]	1.22 [0.94 – 1.60]	1.17 [0.82 – 1.66]	1.20 [0.92 – 1.56]	1.17 [0.82 -1.67]
More brothers	**1.43 [1.10, 1.86] ****	**1.43 [1.10 – 1.86] ****	1.41 [0.99 – 2.00]	**1.50 [1.15 – 1.95] ****	**1.49 [1.05 – 2.12] ***	**1.49 [1.14 – 1.94] ****	**1.55 [1.09 – 2.21] ***
Semi- agriculture and semi-pastoralism	1.04 [0.85 - 1.29]	1.04 [0.84 – 1.28]	1.05 [0.85 – 1.29]	0.88 [0.67 – 1.15]	1.04 [0.84 – 1.28]	0.89 [0.67 – 1.16]	0.88 [0.68 – 1.16]
Pastoralism	1.24 [0.83 -1.86]	1.24 [0.83 – 1.87]	1.24 [0.83 – 1.86]	**0.50 [0.26 – 0.96] ***	1.24 [0.82 -1.86]	**0.48 [0.25 – 0.93] ***	**0.48 [0.25 – 0.92] ***
Non-traditional industry	**1.48 [1.11 – 1.97] ****	**1.44 [1.08 – 1.92] ***	**1.49 [1.12 – 1.99] ****	0.95 [0.65 – 1.39]	**1.44 [1.08 – 1.92] ***	1.15 [0.77 – 1.71]	1.15 [0.77 – 1.71]
[1956-1965] : male		1.23 [0.58 – 2.60]			1.22 [0.58 – 2.59]	1.21 [0.57 – 2.57]	1.20 [0.56 – 2.56]
[1966-1975] : male		1.18 [0.62 – 2.27]			1.18 [0.61 – 2.26]	1.12 [0.58 – 2.16]	1.11 [0.57 – 2.15]
[1976-1985] : male		1.80 [0.94 – 3.45]			1.78 [0.93 – 3.42]	1.63 [0.84 – 3.14]	1.61 [0.83 – 3.12]
[1986-1995] : male		**2.07 [1.10 – 3.93] ***			**2.07 [1.09 – 3.92] ***	1.94 [1.00 – 3.78]	1.92 [0.99 – 3.74]
[>1995] : male		**3.92 [1.84 – 8.36] *****			**3.93 [1.84 – 8.40] *****	**3.60 [1.59 – 8.15] ****	**3.54 [1.56 – 8.04] ****
More sisters : male			1.15 [0.69 – 1.92]		0.99 [0.58 – 1.68]		1.07 [0.63 – 1.82]
More brothers : male			1.03 [0.61 – 1.72]		0.91 [0.53 – 1.54]		0.90 [0.53 -1.53]
Semi- agriculture and semi-pastoralism : male				**1.58 [1.03 – 2.42] ***		1.53 [0.99 – 2.36]	**1.55 [1.00 – 2.39] ***
Pastoralism : male				**5.62 [2.41 – 13.10] *****		**5.98 [2.55 – 14.01] *****	**6.11 [2.60 – 14.35] *****
Non-traditional industry : male				**2.63 [1.55 – 4.46] *****		1.71 [0.95 – 3.08]	1.75 [0.97 – 3.16]
Random effects	**variance (s.d.)**	**variance (s.d.)**	**variance (s.d.)**	**variance (s.d.)**	**variance (s.d.)**	**variance (s.d.)**	**variance (s.d.)**
Mother ID	<0.001 (<0.001)	<0.001 (<0.001)	<0.001 (<0.001)	<0.001 (<0.001)	<0.001 (<0.001)	<0.001 (<0.001)	<0.001 (<0.001)

The column values were OR (Odds Ratio) [95% CI (95% confidence intervals)]. Statistical significance indicated in bold. *$p < 0.05$, **$p < 0.01$, ***$p < 0.001$.

Table S6: Compare sex difference when fixing the cohorts from Model 10, 13, 14 and 15. Other variables are averaged over the levels of sibling configuration and subsistence system.

Comparison	Model 10	Model 13	Model 14	Model 15
**Cohort = “<=1955”: male - female**	**0.382 [0.229, 0.637] *****	**0.386 [0.229, 0.650] *****	**0.554 [0.319, 0.964] ***	**0.562 [0.320, 0.986] ***
**Cohort = “1956 - 1965”: male - female**	**0.469 [0.271, 0.813] ****	**0.472 [0.271, 0.820] ****	0.670 [0.373, 1.200]	0.675 [0.376, 1.213]
**Cohort = “1966 - 1975”: male - female**	**0.452 [0.301, 0.677] *****	**0.454 [0.302, 0.682] *****	**0.620 [0.398, 0.968] ***	**0.625 [0.399, 0.978] ***
**Cohort = “1976 - 1985”: male - female**	**0.688 [0.461, 1.026] •**	0.689 [0.461, 1.028] •	0.903 [0.584, 1.397]	0.906 [0.585, 1.402]
Cohort = “1986 - 1995”: male - female	0.792 [0.541, 1.160]	0.800 [0.538, 1.189]	1.077 [0.703, 1.648]	1.081 [0.698, 1.673]
Cohort = “>1995”: male - female	1.498 [0.858, 2.617]	1.518 [0.850, 2.712]	**1.994 [1.077, 3.691] ***	**1.989 [1.056, 3.747] ***

The column values were OR (Odds Ratio) [95% CI (95% confidence intervals)]. Statistical significance indicated in bold. *$p < 0.05$, **$p < 0.01$, ***$p < 0.001$.

Table S7: Compare sex difference when fixing sibling configuration from Model 11, 13 and 15. Other variables are averaged over the levels of cohorts and subsistence system

Comparison	Model 11	Model 13	Model 15
**The number of siblings = “equal number”: male - female**	**0.583 [0.379, 0.899] ***	**0.664 [0.422, 1.043] •**	0.891 [0.551, 1.442]
**The number of siblings = “more sisters”: male - female**	**0.672 [0.508, 0.889] ******	**0.659 [0.494, 0.879] ******	0.952 [0.676, 1.339]
**The number of siblings = “more brothers”: male - female**	**0.599 [0.449, 0.798] *********	**0.602 [0.448, 0.808] *********	0.801 [0.572, 1.122]

The column values were OR (Odds Ratio) [95% CI (95% confidence intervals)]. Statistical significance indicated in bold. *$p < 0.05$, **$p < 0.01$, ***$p < 0.001$.

Table S8: Compare sex difference when fixing subsistence system from Model 12, 14 and 15. Other variables are averaged over the levels of cohorts and sibling configuration.

Comparison	Model 12	Model 14	Model 15
**Subsistence = "agriculture": male - female**	**0.395 [0.288, 0.540] *** **	**0.439 [0.318, 0.607] *** **	**0.435 [0.311, 0.609] *** **
**Subsistence = "semi-agriculture and semi-pastoralism": male - female**	**0.624 [0.466, 0.834] ** **	**0.672 [0.495, 0.914] * **	**0.674 [0.492, 0.926] * **
**Subsistence = "pastoralism": male - female**	**2.218 [1.010, 4.871] * **	**2.628 [1.183, 5.836] * **	**2.661 [1.196, 5.923] * **
Subsistence = "non-traditional industry": male - female	1.038 [0.678, 1.589]	0.753 [0.470, 1.206]	0.764 [0.472, 1.237]

The column values were OR (Odds Ratio) [95% CI (95% confidence intervals)]. Statistical significance indicated in bold. *$p < 0.05$, **$p < 0.01$, ***$p < 0.001$.

Table S9: Descriptive data for variables using in gifts received models. The full generalized estimating equations (Poisson) predict gifts received in dyads (ego–alter pairs) within the same township in different study years.

Variables			Study-1 (2015) (*Ego* = 188, *Alter* = 2223, *Dyad* = 417263)			Study-2 (2021) (*Ego* = 73, *Alter* = 2323, *Dyad* = 169281)			Variable description
			N (%)	Mean (SD)	Range	N (%)	Mean (SD)	Range	
Ego	Age		188 (100)	44.98 (11.11)	(20, 75)	73 (100)	41.41 (15.19)	(19, 76)	Ego's age at time of Study-1 (2015) or Study-2 (2021)
	Gender	Female	96 (51.06)			54 (73.97)			Ego's gender
		Male	92 (48.94)			19 (26.03)			
	Dispersal pattern	Philopatry	148 (78.72)			42 (57.53)			Ego's dispersal pattern after marriage, being defined by whether staying at natal community
		Dispersal	40 (21.28)			31 (42.47)			
Alter	Age		2223 (100)	43.82 (14.80)	(18, 94)	2323 (100)	46.86 (14.85)	(19, 95)	Alter's age at time of Study-1 (2015) or Study-2 (2021)
	Gender	Female	1162 (52.27)			1213 (52.22)			Alter's gender
		Male	1061 (47.73)			1110 (47.78)			
	Dispersal pattern	Philopatry	1488 (66.94)			1551 (66.77)			Alter's dispersal pattern after marriage, being defined by whether staying at natal community
		Dispersal	735 (33.06)			772 (33.23)			

Table S10: Compare sex difference when fixing study time from gifts received models. Other variables are averaged over the levels of the else variables.

Comparison	Estimate	SE	IRR [95% CI]	*P*
**Study time = "2015": male - female**	**-0.692**	**0.153**	**0.501 [0.371, 0.676]**	**<0.001*** **
Study time = "2021": male - female	-0.181	0.201	0.834 [0.562, 1.237]	0.367

Statistical significance indicates in bold, IRR stood for Incidence Rate Ratio. 95% CI stood for 95% confidence interval. *$p < 0.05$, **$p < 0.01$, ***$p < 0.001$.

Methods S1: Proportion of parents' main livelihood form (when interviewees were unmarried)

Cohort	Subsistence	Number	Proportion
<=1955	Farming	173	0.534
<=1955	Half farming and half pastoral	125	0.386
<=1955	Pastoralism	23	0.071
<=1955	Non-traditional industry	3	0.009
1956-1965	Farming	138	0.517
1956-1965	Half farming and half pastoral	96	0.360
1956-1965	Pastoralism	19	0.071
1956-1965	Non-traditional industry	14	0.052
1966-1975	Farming	210	0.396
1966-1975	Half farming and half pastoral	248	0.468
1966-1975	Pastoralism	34	0.064
1966-1975	Non-traditional industry	38	0.072
1976-1985	Farming	167	0.352
1976-1985	Half farming and half pastoral	225	0.475
1976-1985	Pastoralism	40	0.084
1976-1985	Non-traditional industry	42	0.089
1986-1995	Farming	131	0.302
1986-1995	Half farming and half pastoral	151	0.348
1986-1995	Pastoralism	13	0.030
1986-1995	Non-traditional industry	139	0.320
>1995	Farming	44	0.215
>1995	Half farming and half pastoral	42	0.205
>1995	Pastoralism	0	0.000
>1995	Non-traditional industry	119	0.580

Methods S2: Proportion of income from agriculture, pastoralism, and non-traditional industry across years

Year	Total income	Income	Proportion	Sector
2002	1885.26	381.41	0.20	Agriculture
2003	1849.20	433.00	0.23	Agriculture
2004	1882.20	427.00	0.23	Agriculture
2005	1971.00	424.00	0.22	Agriculture
2006	2091.00	480.00	0.23	Agriculture
2007	2196.00	429.00	0.20	Agriculture
2008	3010.66	504.50	0.17	Agriculture
2009	3627.79	559.84	0.15	Agriculture
2010	4099.04	579.25	0.14	Agriculture
2011	5147.45	605.50	0.12	Agriculture
2012	6134.68	858.00	0.14	Agriculture
2013	7142.05	1343.33	0.19	Agriculture
2002	1885.26	874.12	0.46	Pastoralism
2003	1849.20	855.00	0.46	Pastoralism
2004	1882.20	977.00	0.52	Pastoralism
2005	1971.00	1010.00	0.51	Pastoralism
2006	2091.00	1043.00	0.50	Pastoralism
2007	2196.00	1105.00	0.50	Pastoralism
2008	3010.66	1343.30	0.45	Pastoralism
2009	3627.79	1429.58	0.39	Pastoralism
2010	4099.04	1627.46	0.40	Pastoralism
2011	5147.45	1827.05	0.35	Pastoralism
2012	6134.68	1988.60	0.32	Pastoralism
2013	7142.05	1853.03	0.26	Pastoralism
2002	1885.26	413.56	0.22	Non-traditional industry
2003	1849.20	402.20	0.22	Non-traditional industry
2004	1882.20	423.20	0.22	Non-traditional industry
2005	1971.00	487.00	0.25	Non-traditional industry
2006	2091.00	525.00	0.25	Non-traditional industry
2007	2196.00	601.00	0.27	Non-traditional industry
2008	3010.66	947.80	0.31	Non-traditional industry
2009	3627.79	1383.10	0.38	Non-traditional industry
2010	4099.04	1604.09	0.39	Non-traditional industry
2011	5147.45	2423.30	0.47	Non-traditional industry
2012	6134.68	3075.28	0.50	Non-traditional industry
2013	7142.05	3690.35	0.52	Non-traditional industry

The unit of Total income and Income is 10K CNY
